# Is it safe and feasible to use multi-lateral-pores drainage strategy after video-assisted thoracoscopic surgery?

**DOI:** 10.1371/journal.pone.0313176

**Published:** 2024-11-22

**Authors:** Yingxian Dong, Shujun Li, Guowei Che

**Affiliations:** 1 Department of Thoracic Surgery, West China Hospital, Sichuan University, Chengdu, Sichuan, China; 2 Lung Cancer Center, West China Hospital, Sichuan University, Chengdu, Sichuan, China; 3 The Third Affiliated Hospital of Xinxiang Medical University, Xinxiang, Henan, China; European Institute of Oncology: Istituto Europeo di Oncologia, ITALY

## Abstract

**Objectives:**

Evidence-based studies optimizing chest tube management have been conducted to accelerate the recovery process for lung cancer patients after video-assisted thoracoscopic surgery (VATS). This study is to evaluate whether using the multi-lateral pores chest tube can achieve better drainage performance than conventional-lateral-pore drainage.

**Methods:**

Data from patients undergoing VATS were consecutively collected from September 2023 to June 2024. The groups were randomized into two subgroups, which were multi-lateral-pores drainage group (MDG) and conventional-lateral-pore drainage group (CDG). The primary outcomes included chest drainage performance, and the secondary outcomes included postoperative complications (PPCs).

**Results:**

After screening, 228 patients were randomized into two groups, in which 116 patients in MDG and 112 patients in CDG. The daily drainage volume [199.70 (95%CI: 165.19~234.99) mL/d vs 149.43 (95%CI: 120.70~179.21) mL/d, P<0.01] and total drainage volume [342.79 (95%CI: 291.91~392.63) mL vs 272.68 (95%CI: 225.87~322.11) mL, P = 0.04] in the MDG was significantly higher that that in the CDG. The drainage duration in the MDG was also less than that in the CDG [36.41 (95%CI: 32.23~40.72) h vs 51.02 (95%CI: 46.03~56.38) h, P < 0.01]. The incidence of pleural effusion was lower in the MDG when compared with that in CDG (1.7% vs 9.0%, P = 0.04). No differences were found in the other incidences of chest tube—related PPCs, including pneumothorax (12.0% vs 15.2%, P = 0.15) and subcutaneous emphysema (17.2% vs 17.9%, P = 0.35), however.

**Conclusions:**

Based on this single-center analysis, multi-lateral pores chest tube provided better drainage performance after VATS.

## Introduction

To date, the function of chest tubes, particularly the drainage of fluid or air from the pleural cavity, has remained largely unchanged for over 3000 years [1]. The use of chest drainage tubes after thoracic surgery is crucial for the evacuation of air leaks and/or pleural effusions [2]. Recently, fast-track recovery programs have been applied to thoracic surgery to reduce morbidity, the incidence of postoperative complications, and hospital stay [3]. Moreover, evidence-based studies optimizing chest tube management have been conducted to accelerate the recovery process for lung cancer patients after video-assisted thoracoscopic surgery (VATS) [4–7]. However, controversies and confusions still exist regarding (1) the appropriate number of chest tubes to balance drainage and reduce postoperative pain, (2) the suitable size and type of chest tubes to prevent clogging and milking, (3) whether active suction or the use of a digital classification system should be routinely applied to shorten the duration of air leaks, and (4) the appropriate criteria for early chest tube removal in pleural effusions. There are also numerous studies elaborating on these four types of issues [8–13].

Traditional chest drainage tubes are categorized into different sizes based on their diameter, ranging from 8F to 32F [14]. The most commonly used models in our hospital clinical practice are 20F and 28F drainage tubes, in which 20F chest tube was used for VATS and 28F was used for open thoracic surgery, as they can achieve effective and sufficient drainage of gas and liquid without easy clogging [15]. The conventional chest drainage tube is made of silicone, with a lateral hole at the tube’s tip, primarily to prevent blockage and assist in drainage [16]. Some scholars still prefer to place two drainage tubes for patients undergoing lung surgery, especially for those who have undergone upper lobectomy, with the upper chest tube for air evacuation and the lower chest tube for fluid drainage. Although studies have shown no significant difference in the effects between a single tube and double tubes, based on the habits of clinical doctors, many patients still have double chest tubes placed postoperatively, leading to more intense postoperative pain and discomfort [17–19]. In clinical practice, some surgeons will trim the silicone drainage tubes, such as creating lateral pores at the 5cm and 18cm positions of the drainage tube, mainly to ensure that when the tube is placed to a depth of 25cm, the distal lateral hole can vent the top of the chest, and the proximal lateral hole can drain effusions in areas such as the costophrenic angle, thus avoiding the use of two chest drainage tubes. However, this operation has not been confirmed by research, and to our knowledge, there has been no exploration of the clinical application effects of multi-lateral pores drainage. Therefore, we have conducted this prospective randomized controlled study, aiming to fill this gap in research and provide more options for the management of chest drainage tubes.

## Patients and methods

### Ethical review

Prior to submission, this study was licensed with the Chinese Clinical Trial Registry (ChiCTR2000034999). In addition, in accordance with the Declaration of Helsinki, Ethics Committee on Biomedical Research, West China Hospital of Sichuan University and the Chinese Ethics Committee of Registering Clinical Trials approved our clinical research protocol (#341) in June 2022. Patients were informed of the risks associated with different chest tube use and signed a formal informed consent form, and provided informed written consent for the publication of their study data. Besides, according local policy, Ethics Committee on Biomedical Research, West China Hospital of Sichuan University and the Chinese Ethics Committee of Registering Clinical Trials were consented for publication of raw data obtained from study participants.

### Inclusion and exclusion criteria

**Fig 1:**
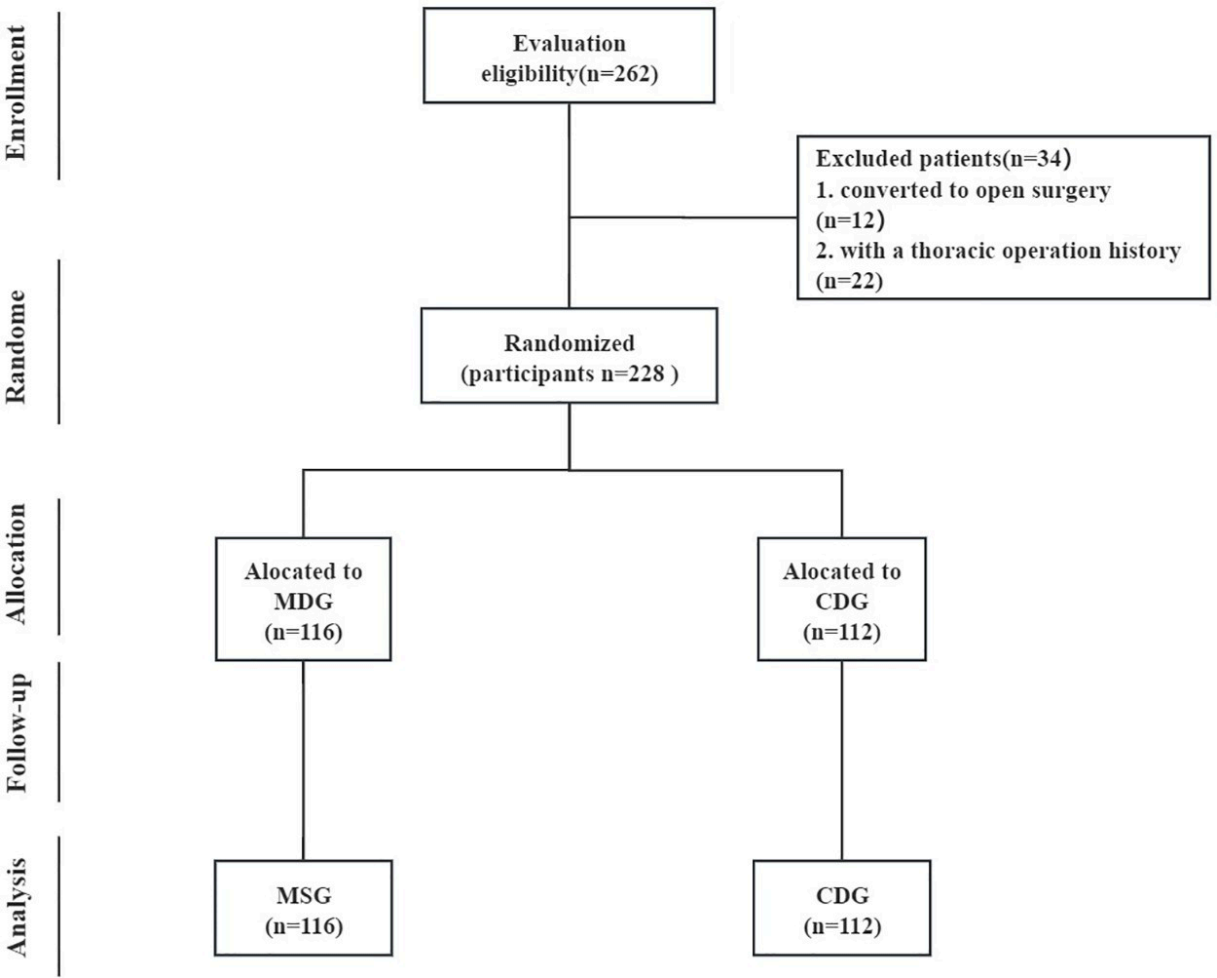
Flow diagram based on consolidated standards of reporting trials.

Patients in the same medical group undergoing surgical treatment for pulmonary nodules in West China Hospital of Sichuan University were collected consecutively. Patients were enrolled if they met the following inclusion criteria: (1) undergoing VATS for pulmonary nodules; (2) American Society of Anesthesiologists (ASA) score of 3 or fewer points. The exclusion criteria were as follows: (1) with a thoracic operation history; (2) converted from VATS to thoracotomy. A CONSORT flow diagram is provided in Fig 1.

### Randomization and blinding

All the patients were randomized into two groups: multi-lateral-pores drainage group (MDG), and conventional-lateral-pore drainage group (CDG). Randomization was based on a computer-generated randomization list, and the results were placed into sequentially numbered, opaque, sealed envelopes by a statistician who did not participate in this clinical trial. The envelope was opened on the day of the operation, and the corresponding tubes were prepared by a surgery nurse who was not involved in patient care. The patients, outcome assessors, and data collectors were blinded to the allocations. The surgeon who performed the VATS could not be blinded, but he did not take part in the data collection of this trial.

### Surgical approach

VATS was mainly performed via the three-portal thoracoscopic technique and double-lumen endotracheal intubation, combined with intravenous anesthesia and single lung ventilation [20]. The thoracoscopy entrance was selected to be 1.5 cm in the 7th intercostal space anterior to the midaxillary line; the main operation port was in the 3rd or 4th intercostal space; and the auxiliary operation port was located at the 9th intercostal space behind the axillary line. When performing systemic lymph node dissection, the left nodes were dissected in groups 5, 6, 7, 8, 9 and 10, and the right nodes were dissected in groups 2, 4, 7, 8, 9 and 10.

### Chest tube management

**Fig 2:**
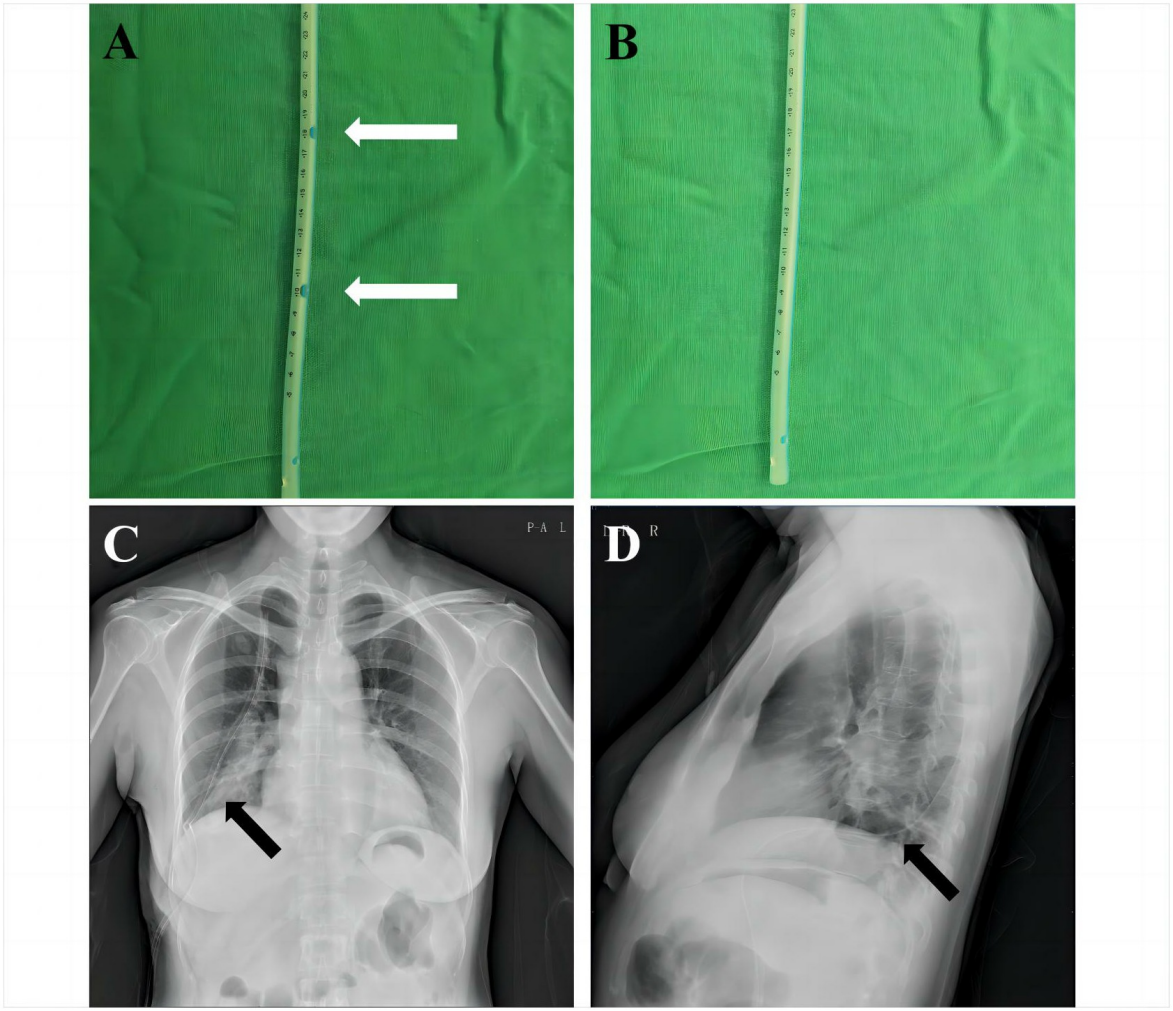
Chest tube management. (A) In the MDG, lateral holes were created at the 5 cm and 18 cm marks of the drainage tube using tissue scissors. (B) In the CDG, the chest tube was not trimmed. As shown in chest X-ray, (C) the tube was inserted from the 7^th^ intercostal space anterior to the midaxillary line, (D) advanced from the posterior mediastinum to the top of the chest cavity, with a depth of 25 cm.

Both groups of patients were fitted with a single 20F silicone chest drainage tube (No.20172140841, Jiangsu Huafei Medical Technology Co., LTD, China). The drainage tube was positioned at the seventh intercostal space along the anterior axillary line, no matter it’s an upper lobectomy or a lower lobectomy. Under the assistance of thoracoscopy, the tube was advanced from the posterior mediastinum to the top of the chest cavity, with a depth of 25 cm (Fig 2). In the MDG, lateral holes were created at the 5 cm and 18 cm marks of the drainage tube using tissue scissors (Fig 2). The trimmed side hole is the same diameter as the normal side hole to ensure that the thoracic drainage tube does not bend or break excessively. A three-cavity water-sealed bottle (No.20152140801, Ningbo Kanghong Medical Equipment Co., LTD, China) was used for chest drainage, and negative pressure was applied after surgery only if persistent air leakage of severe subcutaneous emphysema occurred.

On the first postoperative day, chest X-rays were performed for both groups of patients. The criteria for chest tube withdrawal were: 1) well lung re-expansion confirmed by X-rays imaging, 2) no air leakage was found after surgery, 3) no significant pleural effusion (<300 mL) was shown in X-ray imaging, and 4) the daily serous effusion was under 450 mL/24 h [21].

### Endpoints for the study

The primary outcomes included chest drainage performance, and the secondary outcomes included postoperative complications (PPCs). The chest drainage performance included: (1) daily drainage volume (mL/d): the volume of chest drainage per day, (2) drainage duration (h): the total hours from chest tube insert to chest tube removal, (3) total drainage volume (mL), and (4) length of stay (LOS) after surgery: the number of days accounted from operation to discharge. If any PPCs were identified, then they were recorded as the secondary endpoints for this study and they mainly included: (1) pneumothorax: chest X-ray showing that pleural space was occupied by air (30% of the lungs are compressed); (2) pleural effusion: chest X-ray showing moderate to large fluid accumulation (≥300 mL); (3)subcutaneous emphysema: confirmed by chest X-ray or physical examination; (4)hoarseness; (5)pulmonary infection:clear etiological evidence, imaging showing atelectasis or large patches, fever, and total number of white blood cells >10,000/mL; (6) prolonged air leak (PAL): air leak that persists for more than 5 days postoperatively; and (7) chylothorax: chylous test (+) and daily drainage volume >500 ml; and (8)hemorrhage: more than 200 ml/h of postoperative bloody drainage fluid that lasts for 3 h. PPC criteria were according to the STS/ESTS (2015) complication definitions [22].

### Statistical analysis

Since this study is the first to assess the effect of multi-lateral-pores drainage on drainage performance, we based our sample size calculation on the assumption that a mean drainage duration of 10 hours lower in MDG than that CDG (with standard deviation = 10 hours) is clinically highly relevant. One hundred patients per group provided 90% power with a 2-sided α of 5% to detect a significant difference in drainage duration between the groups. Considering a dropout rate of 10%, 110 patients were required in each group.

Demographic data collected were recorded as follows: the means and SDs represented continuous data, medians and ranges represented non-normally distributed data, and proportions were represented by binary variables. The Student’s t-test and the Mann–Whitney U-test were used to make comparisons. In the case of categorical data, the chi-squared test or the Fisher’s exact test was used to perform the comparisons. P < 0.05 (two-tailed) was found to be statistically significant in all of the analyses. All the statistical analyses were conducted by using the Statistical Package for the Social Sciences (SPSS) software (version 26.0, IBM Corporation, Armonk, New York, USA), which was used to analyze the data.

## Results

### Participants

From September 2023 to June 2024, 262 participants were assessed for eligibility for the trial. A total of 34 participants were excluded before randomization, with the remaining 228 participants randomized into two groups, including 116 patients in the MDG and 112 patients in the CDG. The clinical characteristics, pulmonary function, histology, and TNM stage [2023 union for international cancer control (UICC)], which were matched and comparable, are listed in Table 1.

**Table 1:** Population characteristics in two groups.

Index			MDG (n = 116)	CDG (n = 112)
Gender	Male		44 (37.9%)	52(46.4%)
	Female		72(62.1%)	60(53.6%)
Age (year)			58.10(32–84)	55.86(30–73)
BMI (kg/m^2^)			23.19(17.96–27.94)	23.16(17.63–30.76)
Smoking history	Yes		34(29.3%)	30(26.8%)
	No		82(70.7%)	82(73.2%)
Pulmonary function	FEV1		2.90(1.67–5.20)	2.84(1.67–4.12)
	FEV1/FVC, %		83.24(61.95–99.27)	82.25(49.00–99.27)
Comorbidities	COPD		18(15.5%)	18(16.1%)
	Hypertension		26(22.4%)	16(14.3%)
	Diabetes		6(5.2%)	8(7.1%)
	CHD		10(8.6%)	4(3.6%)
Operation approach	Lobectomy		42(36.2%)	44(39.3%)
	Segmentectomy		59(50.9%)	54(48.2%)
	Wedge resection		15(12.9%)	14(12.5%)
Duration (min)	Surgery		82.16(53.00–190.00)	87.95(37.00–198.00)
	Anesthesia		114.16(83.00–220.00)	117.95(67.00–228.00)
Blood loss (mL)			14.83(5.00–110.00)	11.16(5.00–40.00)
ASA score	I		0	0
	II		98(84.5%)	102(91.1%)
	III		18(15.5%)	10(8.9%)
Histology	Adenocarcinoma		90(77.6%)	86(76.8%)
	Squamouscarinoma		6(5.2%)	12(10.7%)
	Benign		16(13.8%)	12(10.7%)
	Others		4(3.4%)	2(1.8%)
TNM stage (2023 UICC)	T	T1	88(75.9%)	92(82.1%)
		T2	8(6.9%)	6(5.4%)
		T3	0	0
	N	N0	90(77.6%)	98(87.5%)
		N1	6(5.2%)	0
		N2	0	0
	M	M0	96(82.8%)	98(87.5%)
		M1	0	0

Abbreviation: MDG: Multi-lateral-pores drainage group; CDG: Conventional-lateral-pore drainage group; BMI: Body mass index; FEV1: Forced expiratory volume in the first second; FVC: Forced vital capacity; COPD: Chronic obstructive pulmonary disease; CHD: Coronary heart disease; ASA: American society of aneshesiologists.

### Surgery-related outcome

Since intraoperative conditions, surgical sites, and surgical methods may affect the performance of postoperative thoracic drainage, we compared the surgery-related outcomes of the two groups of patients. By comparison, we found that there was no statistical difference in the proportion of pleural adhesions (P = 0.47), operation method (P = 0.34) and operation site (P = 0.71) between the two groups, in which details can be seen in Table 2. Besides, S1 and S2 Tables showed the subgroup analyses of drainage performance between different surgical sites and drainage performance in whether with pleural adhesion.

**Table 2:** Surgery-related characteristics in two groups.

Index		MDG (n = 116)	CDG (n = 112)	P value
Operation approach	Lobectomy	42(36.2%)	44(39.3%)	0.34
	Segmentectomy	59(50.9%)	54(48.2%)	
	Wedge resection	15(12.9%)	14(12.5%)	
surgical site	LUL	15(12.9%)	14(12.5%)	0.71
	LLL	23(19.8%)	19(17.0%)	
	RUL	30(25.9%)	28(25.0%)	
	RML	22(19.0%)	17(15.2%)	
	RLL	26(22.4%)	34(30.45%)	
Pleural adhesions	None	99(85.3%)	94(83.9%)	0.47
	Local	11(9.5%)	8(7.1%)	
	Extant	6(5.2%)	10(8.9%)	
Duration (min)	Surgery	82.16(53.00–190.00)	87.95(37.00–198.00)	0.25
	Anesthesia	114.16(83.00–220.00)	117.95(67.00–228.00)	0.25
Blood loss (mL)		14.83(5.00–110.00)	11.16(5.00–40.00)	0.09

Abbreviation: MDG: Multi-lateral-pores drainage group; CDG: Conventional-lateral-pore drainage group; RUL: Right upper lobe; RML: Right middle lobe; RLL: Right lower lobe; LUL: Left upper lobe; LLL: Left lower lobe.

### Drainage performance

As shown in Table 3, the daily drainage volume [199.70 (95%CI: 165.19~234.99) mL/d vs 149.43 (95%CI: 120.70~179.21) mL/d, P< 0.01] and total drainage volume [342.79 (95%CI: 291.91~392.63) mL vs 272.68 (95%CI: 225.87~322.11) mL, P = 0.04] in the MDG was significantly higher that that in the CDG. However, the drainage duration in the MDG was less than that in the CDG [36.41 (95%CI: 32.23~40.72) h vs 51.02 (95%CI: 46.03~56.38) h, P < 0.01]. Besides, LOS after surgery l3.22 (95%CI: 3.84~4.22) d vs 3.45 (95%CI: 3.99~4.42) d, P = 0.31] between two groups were comparable.

**Table 3:** Drainage performance between two groups.

Index	MDG(n = 116)	CDG(n = 112)	P value
Daily drainage volume (mL/d)	199.70 (95%CI: 165.19~234.99)	149.43 (95%CI: 120.70~179.21)	0.03
Drainage duration (h)	36.41 (95%CI: 32.23~40.72)	51.02 (95%CI: 46.03~56.38)	< 0.01
Total drainage volume (mL)	342.79 (95%CI: 291.91~392.63)	272.68 (95%CI: 225.87~322.11)	0.04
LOS after surgery (d)	3.22 (95%CI: 3.84~4.22)	3.45 (95%CI: 3.99~4.42)	0.31

Abbreviation: MDG: Multi-lateral-pores drainage group; CDG: Conventional-lateral-pore drainage group; LOS: Length of stay; CI: Confidence interval.

### Postoperative complications

The total incidence of PPCs was found comparable between the MDG and CDG (36.2% vs 45.5%, P = 0.09). Interestingly, the incidence of pleural effusion was lower in the MDG when compared with that in CDG (1.7% vs 9.0%, P = 0.04). No differences were found in the other incidences of chest tube—related PPCs, including pneumothorax (12.0% vs 15.2%, P = 0.15) and subcutaneous emphysema (17.2% vs 17.9%, P = 0.35), however. The difference in the incidence of pulmonary infection (3.4% vs 1.8%, P = 0.43) and severe pain (1.7% vs 1.8%, P = 0.88) between the MDG and the CDG was insignificant. Details can be seen in Table 4.

**Table 4:** Postoperative complications between two groups.

	MDG(n = 116)	CDG(n = 112)	P value
Pleural effusion	2(1.7%)	10(8.9%)	0.04
pneumothorax	14(12.1%)	17(15.2%)	0.15
Subcutaneous emphysema	20(17.2%)	20(17.9%)	0.35
Pulmonary infection	4(3.4%)	2(1.8%)	0.43
Chylothorax	0	0	
Persistent air leakage	0	0	
Hemorrhage	0	0	
Hoarse	0	0	
Atelectasis	0	0	
Severe Pain	2(1.7%)	2(1.8%)	0.88
Total PPCs	42(36.2%)	51(45.5%)	0.09

Abbreviation: MDG: Multi-lateral-pores drainage group; CDG: Conventional-lateral-pore drainage group. PPC: Postoperative complications.

## Discussion

In this prospective, randomized, controlled study, the researchers found that multi-lateral-pores drainage strategy may achieve better drainage performance, such as daily drainage volume and drainage duration, when compared with conventional-lateral-pore drainage strategy. Meanwhile, the lower incidence of pleural effusion was found in MDG.

The concept of enhanced recovery after surgery (ERAS), advancements in surgical techniques, and the development of medical devices have led to increased attention from thoracic surgeons on the rapid recovery and improved quality of life for lung cancer patients postoperatively. Thoracic drainage management is an essential part of perioperative management and a crucial step in the process of rapid postoperative recovery. In recent years, researchers have conducted multifaceted discussions and studies on the type, size, and number of chest tubes, as well as suction, digital system, and chest tube milking [23]. All discussions were aimed to better drainage performance to enhance patients recovery. In our study, it’s the first time to discuss the effect of lateral pores on drainage performance, which have shown the ideal outcomes.

In our study, better daily drainage volume and duration were found in MDG. Like all cylindrical structures, chest tubes abide by the physics of Poiseuille’s law and the Fanning equation, and the daily drainage volume is responsible for drainage velocity [24]. The conventional-lateral-pore chest tube was insert to the top of thorax, aimed to evacuation of pleural cavity air accumulation. However, in this situation, the drainage of pleural effusion is significantly influenced by the patient’s position, which decreased the drainage velocity. When inserted with multi-lateral-pores chest tube, the pleural effusion can be pull out from pores of different heights, without the limitation of patient position and thoracic pressure changes, thus realizing the efficient drainage.

Traditionally, thoracic surgeons have used two chest tubes to drain the pleural space after lobectomy, aimed to drain effusion by the lower tube and drain gas by the upper tube. Several randomized trials have demonstrated that the use of a single chest tube after lobectomy is safe and effective with no differences in residual pleural effusion or the need to reinsert a chest tube but is significantly less painful than two drains [17–19]. Furthermore, a single drain is associated with a reduced duration of chest drainage and a smaller volume of fluid drained [19]. In our study, the multi-lateral-pores drainage was inserted from the 7^th^ intercostal space and extended from the posterior mediastinum to the top of the chest, which can achieve ideal effusion drainage by multi-lateral pores of the chest tube and gas discharge by the upper lateral pore of the chest tube. However, different from other studies, MDG was found a increased daily drainage volume but decreased drainage duration, in which we think it’s because of the more efficient drainage achieved by multi-lateral pores. In MDG, whenever pleural effusion occurs, the multi-lateral hole drainage tube can always drain pleural effusion earlier, while in CDG, patients often need to turn over and change their body position to assist drainage. In this case, the MDG can achieve a higher daily drainage volume within the same time. However, the patients in the CDG did not completely drain the newly generated pleural effusion every day, so the daily drainage volume was lower than MDG. Correspondingly, the MDG reached the chest tube removal standard earlier (no obvious pleural effusion), while the CDG did not achieve effective drainage, still had pleural effusion and did not meet the tube removal standard. Accordingly, drainage duration was prolonged, and the proportion of patients with pleural effusion increased.

Other newly type of chest tubes, like the Smart Drain Coaxial (SDC) chest tube and Blake drainage (BD) tube, have been discussed recently [25–27]. The SDC chest tube, is built with an internal lumen with distal bores for air evacuation and four external fluted channels for fluid drainage [25]. In Bassi et al. Study, the 28 F SDC chest tube showed lower pain and shorter LOS but no difference in drainage performance when compared with standard chest tube management (one upper 28-Fr and one lower 32-Fr standard chest tube) after open pulmonary lobectomy. The BD tube is equipped with four longitudinal grooves to avoid occlusion and allow efficient drainage, which was widely used in cardiac surgery [28] and now has often been used in minimal invasive thoracic surgery [27]. However, suction is required for the BD to achieve sufficient air evacuation, since it can be easily clogged with cellulose exudates [29]. As discussed in our study, because only one 20F multi-lateral-pores chest tube is needed, the patients’ pain was greatly reduced. And different from BD tube or SDC tube, there is no other intra-caluminal structure of the multi-lateral-pores chest tube, which greatly reduces the possibility of drainage tube blockage. Of course, the comparison of the drainage effect of these types of drainage tubes still needs to be confirmed by further research.

Although the results confirmed that multi-lateral-pores drainage could effectively improve drainage performance after VATS, this study has limitations. First, we only discussed the feasibility of multi-lateral-pores drainage in three-ports VATS, these results may be limited in open surgery or one-port VATS. Future study will focus on the other types of thoracic surgery. Second, this study was conducted in a single center, and all operations were performed by the same proficient surgeon who had completed more than 3000 VATS and was proficient. Therefore, it is not clear whether the surgical technique will affect the results of the study and whether other surgeons can repeat the results. Third, due to the surgeon preference and institutional differences in chest drainage programs, we only studied the 20F Silicone chest tube for drainage. Additional chest tubes, such as 18F or 28F Silicone chest tube, Blake Drain, and pigtail catheter, will be the focus of future research. Last, the multi-lateral-pores chest tube was tailored with additional holes created with scissors. This procedure, altering the manufactured product, may be not appropriate in terms of patients’ safety and quality controls. Though no drainage tube fracture has ever occurred in the clinic, but this is also one of the limitations of this study, and specially produced multi-lateral-pores tubes may be needed for future research.

## Conclusions

Based on this single-center analysis, multi-lateral pores chest tube provided better drainage performance than conventional-lateral-pore chest tube after VATS, without increasing the rates of PPCs.

## Supporting information

S1 Checklist CONSORT 2010 checklist of information to include when reporting a randomised trial*. (DOC)

S1 Table Surgical site subgroup analyses of drainage performance. (DOCX)

S2 Table Pleural adhesion subgroup analyses of drainage ferformance. (DOCX)

S1 File (DOCX)

S1 Data (XLSX)
