# Supplementary material for: Is it safe and feasible to use multi-lateral-pores drainage strategy after video-assisted thoracoscopic surgery?
Source: PLoS One. 2024 Nov 22;19(11):e0313176. doi: 10.1371/journal.pone.0313176 (PMC11584125; doi:10.1371/journal.pone.0313176)
Supplement: S1 Table — (DOCX) [file pone.0313176.s002.docx]

| Supplementary Table 1. Surgical site Subgroup analyses of drainage performance | | | | | | |
| --- | --- | --- | --- | --- | --- | --- |
| Index | MDG(n=116) | | P value | CDG(n=112) | | P value |
|  | LLSG(n=49) | ULSG(n=67) |  | LLSG(n=53) | ULSG(n=59) |  |
| Daily drainage volume (mL/d) | 280.65 ± 237.69 | 140.50 ± 98.75 | 0.169 | 176.71 ± 186.13 | 124.94 ± 124.93 | 0.084 |
| Drainage duration (h) | 39.32 ± 25.47 | 33.64 ± 18.81 | 0.314 | 53.27 ± 35.88 | 49.91 ± 16..68 | 0.546 |
| Total drainage volume (mL/d) | 449.37 ± 291.94 | 264.85 ± 215.81 | < 0.001 | 320.36 ± 299.50 | 229.85 ± 216.18 | 0.073 |
| LOS after surgery (d) | 3.03 ± 0.91 | 4.16 ± 1.15 | 0.09 | 3.31 ± 1.52 | 3.59 ± 0.87 | 0.356 |
| Abbreviation: MDG: multi-lateral-pores drainage group; CDG: conventional-lateral-pore drainage group; LOS: length of stay. LLSG: Lower lobe subgroup; ULSG: Upper lobe subgroup (which include right middle lobe). | | | | | | |
