# Supplementary material for: Is it safe and feasible to use multi-lateral-pores drainage strategy after video-assisted thoracoscopic surgery?
Source: PLoS One. 2024 Nov 22;19(11):e0313176. doi: 10.1371/journal.pone.0313176 (PMC11584125; doi:10.1371/journal.pone.0313176)
Supplement: S2 Table — (DOCX) [file pone.0313176.s003.docx]

| Supplementary Table 2. Pleural adhesion Subgroup analyses of drainage performance | | | | | | |
| --- | --- | --- | --- | --- | --- | --- |
| Index | MDG(n=116) | | P value | CDG(n=112) | | P value |
|  | PASG(n=17) | NPASG(n=99) |  | PASG(n=18) | NPASG(n=94) |  |
| Daily drainage volume (mL/d) | 277.63 ± 212.46 | 186.32 ± 176.94 | 0.059 | 173.16 ± 156.18 | 144.89 ± 159.12 | 0.49 |
| Drainage duration (h) | 37.39 ± 23.50 | 34.20 ± 20.09 | 0.6 | 66.71 ± 32.48 | 48.59 ± 26.81 | 0.013 |
| Total drainage volume (mL/d) | 473.77 ± 358.89 | 320.30 ± 241.72 | 0.027 | 440.56 ± 346.18 | 240.53 ± 230.98 | 0.029 |
| LOS after surgery (d) | 3.93 ± 1.05 | 3.04 ± 1.07 | 0.691 | 4.03 ± 1.33 | 3.03 ± 1.14 | 0.001 |
| Abbreviation: MDG: multi-lateral-pores drainage group; CDG: conventional-lateral-pore drainage group; LOS: length of stay.PAG: Pleural adhesion subgroup; NPAG: Non-pleural adhesion subgoroup. | | | | | | |
