# Supplementary material for: Is it safe and feasible to use multi-lateral-pores drainage strategy after video-assisted thoracoscopic surgery?
Source: PLoS One. 2024 Nov 22;19(11):e0313176. doi: 10.1371/journal.pone.0313176 (PMC11584125; doi:10.1371/journal.pone.0313176)
Supplement: S1 File — (DOCX) [file pone.0313176.s004.docx]

**Safety and feasibility of multi-lateral-pores drainage after video-assisted thoracoscopic surgery: A a protocol for a prospective, randomized, controlled, observer-blinded study.**

**Introduction**

To date, the function of chest tubes, particularly the drainage of fluid or air from the pleural cavity, has remained largely unchanged for over 3000 years[1]. The use of chest drainage tubes after thoracic surgery is crucial for the evacuation of air leaks and/or pleural effusions[2]. Recently, fast-track recovery programs have been applied to thoracic surgery to reduce morbidity, the incidence of postoperative complications, and hospital stay[3]. Moreover, evidence-based studies optimizing chest tube management have been conducted to accelerate the recovery process for lung cancer patients after video-assisted thoracoscopic surgery (VATS)[4-7]. However, controversies and confusions still exist regarding (1) the appropriate number of chest tubes to balance drainage and reduce postoperative pain, (2) the suitable size and type of chest tubes to prevent clogging and milking, (3) whether active suction or the use of a digital classification system should be routinely applied to shorten the duration of air leaks, and (4) the appropriate criteria for early chest tube removal in pleural effusions. There are also numerous studies elaborating on these four types of issues[8-13].

Traditional chest drainage tubes are categorized into different sizes based on their diameter, ranging from 8F to 32F[14]. The most commonly used models in clinical practice are 20F and 28F drainage tubes, as they can achieve effective and sufficient drainage of gas and liquid without easy clogging[15]. The conventional chest drainage tube is made of silicone, with a lateral hole at the tube's tip, primarily to prevent blockage and assist in drainage[16]. Some scholars still prefer to place two drainage tubes for patients undergoing lung surgery, especially for those who have undergone upper lobectomy, with the upper chest tube for air evacuation and the lower chest tube for fluid drainage. Although studies have shown no significant difference in the effects between a single tube and double tubes, based on the habits of clinical doctors, many patients still have double chest tubes placed postoperatively, leading to more intense postoperative pain and discomfort[17-19]. In clinical practice, some surgeons will trim the silicone drainage tubes, such as creating lateral pores at the 5cm and 18cm positions of the drainage tube, mainly to ensure that when the tube is placed to a depth of 25cm, the distal lateral hole can vent the top of the chest, and the proximal lateral hole can drain effusions in areas such as the costophrenic angle, thus avoiding the use of two chest drainage tubes. However, this operation has not been confirmed by research, and to our knowledge, there has been no exploration of the clinical application effects of multi-lateral pores drainage. Therefore, we have conducted this prospective randomized controlled study, aiming to fill this gap in research and provide more options for the management of chest drainage tubes.

**Aim**

The aim of the study is to develop a novel drainage protocol to provide better drainage performance postoperative patients, reduce the length of stay, and accelerate postoperative recovery of patients.

**Objectives and methods**

The study objectives are to:

Determine whether multi-lateral pores drainage strategy would achieve better drainage performance compared with conventional-lateral-pore drainage; shorten the drainage duration and length of stay.

Determine whether multi-lateral pores drainage strategy increased or decreased the rate of postoperative complications (PPCs) compared with conventional-lateral-pore drainage.

**Study design**

A single-center, individually randomized, parallel, controlled trial will be conducted. Participants will give informed consent and will be enrolled in one of two groups: an intervention group, which consists of multi-lateral pores drainage strategy (MDG) ; or control group, conventional-lateral-pore drainage (CDG).

**Setting**

Patients will be recruited from the same medical team at the Department of Thoracic Surgery, West China Hospital, Sichuan University. Both groups of patients were fitted with a single 20F silicone chest drainage tube. The drainage tube was positioned at the seventh intercostal space along the anterior axillary line. Under the assistance of thoracoscopy, the tube was advanced from the posterior mediastinum to the top of the chest cavity, with a depth of 25 cm. In the MDG, lateral holes were created at the 5 cm and 18 cm marks of the drainage tube using tissue scissors. The drainage performance were investigated, including (1) daily drainage volume (mL/d): the volume of chest drainage per day, (2) drainage duration (h): the total hours from chest tube insert to chest tube removal, and (3) length of stay (LOS) after surgery: the number of days accounted from operation to discharge. All PPCs events were recorded in the clinical case report form (CRF) form ( Additional file 1 ).

**Sample size assessment**

Since this study is the first to assess the effect of multi-lateral-pores drainage on drainage performance, we based our sample size calculation on the assumption that a mean daily drainage volume difference of 50 mL (with standard deviation = 10 mL) is clinically highly relevant. With a 5% α risk, a 10% β risk, and a bilateral test, the total number of teams required was 100 (50 per group).

**Recruitment**

The study will recruit 100 participants for surgical treatment of pulmonary nodules in Chengdu, China, recruited and evaluated by a senior thoracic surgeon (Guowei Che) in an outpatient setting.

**Participant inclusion/exclusion criteria**

Patients in the same medical group undergoing surgical treatment for pulmonary nodules in West China Hospital of Sichuan University were collected consecutively. Patients were enrolled if they met the following inclusion criteria: (1) undergoing VATS for pulmonary nodules; (2) American Society of Anesthesiologists (ASA) score of 3 or fewer points. The exclusion criteria were as follows: (1) with a thoracic operation history; (2) converted from VATS to thoracotomy. In addition, we will invite any participants who drop out of the study to be interviewed to explore their reasons for withdraw out.

**Randomization and Intervention**

All the patients were randomized into two groups: an intervention group, which consists of multi-lateral pores drainage strategy (MDG); a control group, conventional-lateral-pore drainage (CDG). Randomization was based on a computer-generated randomization list, and the results were placed into sequentially numbered, opaque, sealed envelopes by a resascher (Shujun, Li) who did not participate in this clinical trial operation procedure.

Under thoracoscopic guidance, the surgeon (Yingxian Dong) inserted the chest tube based on the randomlized outcome. Both groups of patients were fitted with a single 20F silicone chest drainage tube. The drainage tube was positioned at the seventh intercostal space along the anterior axillary line. Under the assistance of thoracoscopy, the tube was advanced from the posterior mediastinum to the top of the chest cavity, with a depth of 25 cm (Figure 1). In the MDG, lateral holes were created at the 5 cm and 18 cm marks of the drainage tube using tissue scissors (Figure 1).


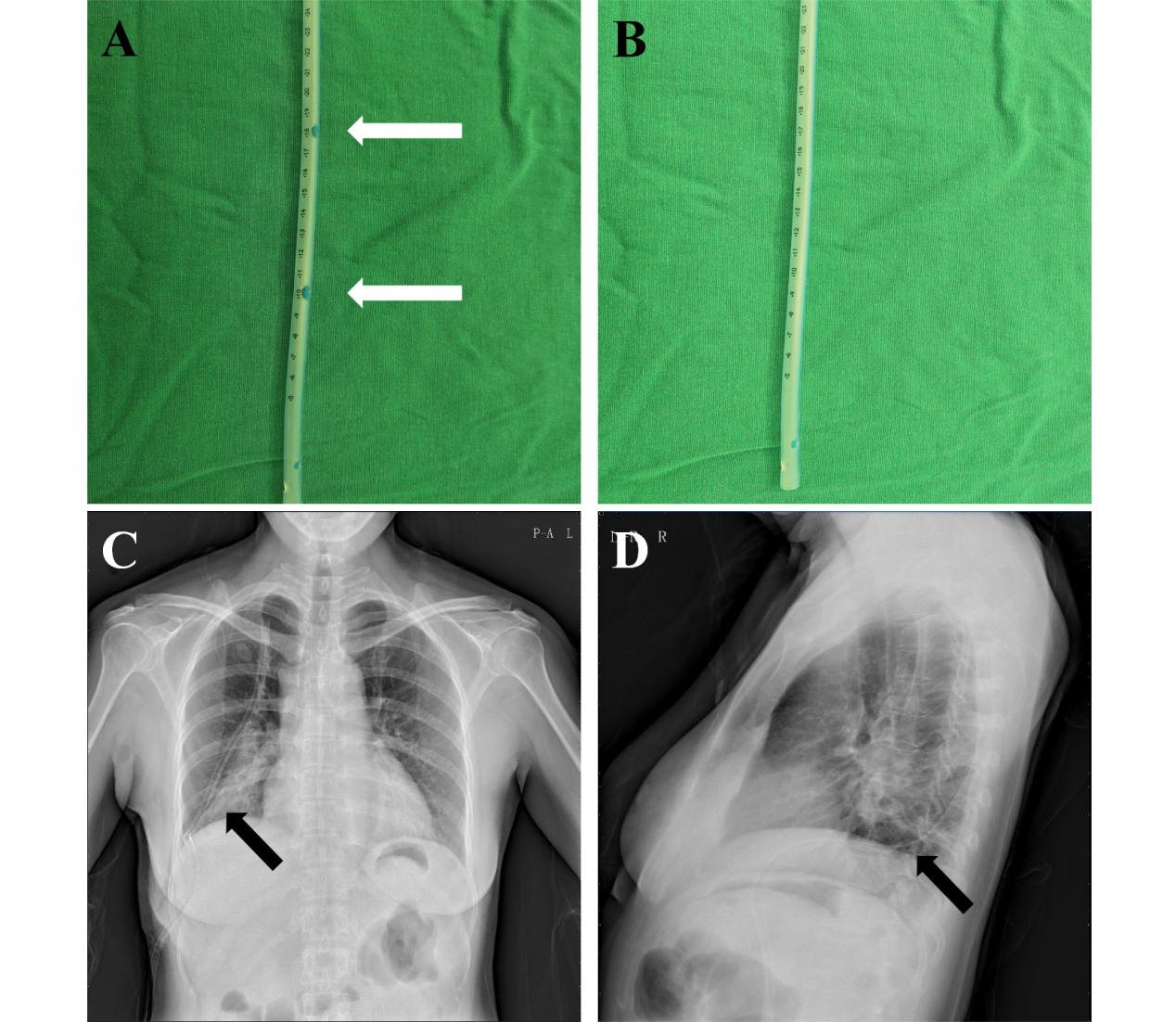


Figure 1. Chest tube management. (A) In the MDG, lateral holes were created at the 5 cm and 18 cm marks of the drainage tube using tissue scissors. (B) In the CDG, the chest tube was not trimmed. As shown in chest X-ray, (C) the tube was inserted from the 7^th^ intercostal space anterior to the midaxillary line, (D) advanced from the posterior mediastinum to the top of the chest cavity, with a depth of 25 cm.

**Blinding**

Blinding is not possible for the implementer to implement the intervention. Because different chest tubes need to be configured in the operating room, the implementor of the intervention (Yingxian Dong) and the designer (Guowei, Che) will inevitably know the drug use of each patient. The blinds that researchers were assigned during the evaluation of results during feasibility studies proved impossible in practice because participants often unintentionally unblinds researchers, a problem that has been reported in similar trials. Therefore, in order to minimize confirmation bias, these two investigators were not involved in the evaluation of study results, collection of clinical baseline data, and analysis of data.

.

**Data collection**

The CRF table is shown in Additional file 1 , and data were collected by one researcher (Shujun Li), he was not involved in the implementation of the intervention.

**Surgical Approach**

The VATS procedure was mainly performed using the three-portal thoracoscopic technique in two groups. The thoracoscopy entrance was selected to be 1.5 cm in the 7th intercostal space anterior to the midaxillary line. The main operation port was in the 3rd or 4th intercostal space, while the auxiliary operation port was placed at the 9th intercostal space behind the axillary line.

**Chest tube removal**

On the first postoperative day, chest X-rays were performed for both groups of patients. The chest tubes were removed after confirming good lung re-expansion, no air leakage, and no significant pleural effusion. The chest tube could be removed safely even if the daily serous effusion was of a high volume (up to 450 mL/24 h)

**Withdrawal criteria**

Participants were withdrawn from the study if they met the following criteria: 1) cancellation of surgery; 2) patients do not withdraw and refuse to follow up; 3) patients who were converted to thoracotomy.

**Outcomes**

The primary outcomes included chest drainage performance, and the secondary outcomes included postoperative complications (PPCs). The chest drainage performance included: (1) daily drainage volume (mL/d): the volume of chest drainage per day, (2) drainage duration (h): the total hours from chest tube insert to chest tube removal, and (3) length of stay (LOS) after surgery: the number of days accounted from operation to discharge. If any PPC were identified, then they were recorded as the secondary endpoints for this study and they mainly included: (1) pneumothorax: chest X-ray showing that pleural space was occupied by air; (2) pleural effusion: chest X-ray showing fluid accumulation; (3)subcutaneous emphysema: confirmed by chest X-ray or physical examination; (4)hoarseness; (5)pulmonary infection:clear etiological evidence, imaging showing atelectasis or large patches, fever, and total number of white blood cells >10,000/mL; (6) prolonged air leak (PAL): air leak that persists for more than 5 days postoperatively; and (7) chylothorax: chylous test (+) and daily drainage volume >500 ml; and (8)hemorrhage: more than 200 ml/h of postoperative bloody drainage fluid that lasts for 3 h. PPC criteria were according to the STS/ESTS (2015) complication definitions

**Measurement**

The baseline data of the patients, the use of anesthetics, and the assessment of symptoms were managed by one study investigator (Shujun Li), who was not involved in the implementation of the intervention or the analysis of the data. Uniform training was conducted before the study to ensure consistency of assessment. Controversial cases were discussed within the research group and consensus was obtained.

**Quality control**

Data will be checked periodically by quality control personnel (Yingxian, Dong and Guowei, Che). Participant privacy information is not recorded in the CRF. Each participant will be assigned a study number that will be used in all study files, which are for the use of the study staff only.

**Chest tube related adverse events**

There may be adverse effects associated with the chest tube, including pain, poor wound healing or obstruction of chest tube.

**Ethics**

All recruited patients were required to provide written informed consent. Any subsequent amendments to the protocol will be submitted for further review and approval. This study was approved by the ethics application of West China Hospital of Sichuan University. The results of this study will be disseminated through peer-reviewed publications and academic conferences.

**Statistics and data analysis**

Data collation and analysis were performed by two researchers (Yingxian Dong and Guowei Che), with additional consulting by a statistician who was not involved in the study as a consultant.

The first 50 randomised participants will constitute the sample for an internal pilot, to check on recruitment and data completion. There will be no other interim analyses.

**References**

1. Miller KS, Sahn SA. Chest tubes. Indications, technique, management and complications. Chest. 1987;91(2):258-264. doi:10.1378/chest.91.2.258
2. Lima VP, Bonfim D, Risso TT, et al. Influence of pleural drainage on postoperative pain, vital capacity and six-minute walk test after pulmonary resection. J Bras Pneumol. 2008;34(12):1003-1007. doi:10.1590/s1806-37132008001200004
3. Batchelor TJP, Rasburn NJ, Abdelnour-Berchtold E, et al. Guidelines for enhanced recovery after lung surgery: recommendations of the Enhanced Recovery After Surgery (ERAS®) Society and the European Society of Thoracic Surgeons (ESTS). Eur J Cardiothorac Surg. 2019;55(1):91-115. doi:10.1093/ejcts/ezy301
4. Ishikura H, Kimura S. The use of flexible silastic drains after chest surgery: novel thoracic drainage. Ann Thorac Surg. 2006;81(1):331-333. doi:10.1016/j.athoracsur.2005.05.102
5. Nakashima S, Watanabe A, Mishina T, Obama T, Mawatari T, Higami T. Feasibility and safety of postoperative management without chest tube placement after thoracoscopic wedge resection of the lung. Surg Today. 2011;41(6):774-779. doi:10.1007/s00595-010-4346-5
6. Watanabe A, Watanabe T, Ohsawa H, et al. Avoiding chest tube placement after video-assisted thoracoscopic wedge resection of the lung. Eur J Cardiothorac Surg. 2004;25(5):872-876. doi:10.1016/j.ejcts.2004.01.041
7. Gocyk W, Kużdżał J, Włodarczyk J, et al. Comparison of Suction Versus Nonsuction Drainage After Lung Resections: A Prospective Randomized Trial. Ann Thorac Surg. 2016;102(4):1119-1124. doi:10.1016/j.athoracsur.2016.04.066
8. Tanaka M, Sagawa M, Usuda K, et al. Postoperative drainage with one chest tube is appropriate for pulmonary lobectomy: a randomized trial. Tohoku J Exp Med. 2014;232(1):55-61. doi:10.1620/tjem.232.55
9. Sakakura N, Fukui T, Mori S, Hatooka S, Yokoi K, Mitsudomi T. Fluid drainage and air evacuation characteristics of Blake and conventional drains used after pulmonary resection. Ann Thorac Surg. 2009;87(5):1539-1545. doi:10.1016/j.athoracsur.2009.02.013
10. Dango S, Sienel W, Passlick B, Stremmel C. Impact of chest tube clearance on postoperative morbidity after thoracotomy: results of a prospective, randomised trial. Eur J Cardiothorac Surg. 2010;37(1):51-55. doi:10.1016/j.ejcts.2009.06.034
11. Cerfolio RJ, Bryant AS. The management of chest tubes after pulmonary resection. Thorac Surg Clin. 2010;20(3):399-405. doi:10.1016/j.thorsurg.2010.04.001
12. Icard P, Chautard J, Zhang X, et al. A single 24F Blake drain after wedge resection or lobectomy: a study on 100 consecutive cases. Eur J Cardiothorac Surg. 2006;30(4):649-651. doi:10.1016/j.ejcts.2006.06.032
13. Refai M, Brunelli A, Varela G, et al. The values of intrapleural pressure before the removal of chest tube in non-complicated pulmonary lobectomies. Eur J Cardiothorac Surg. 2012;41(4):831-833. doi:10.1093/ejcts/ezr056
14. Cooke DT, David EA. Large-bore and small-bore chest tubes: types, function, and placement. Thorac Surg Clin. 2013;23(1):17-v. doi:10.1016/j.thorsurg.2012.10.006
15. Shalli S, Saeed D, Fukamachi K, et al. Chest tube selection in cardiac and thoracic surgery: a survey of chest tube-related complications and their management. J Card Surg. 2009;24(5):503-509. doi:10.1111/j.1540-8191.2009.00905.x
16. Demmy TL, Nwogu C, Solan P, Yendamuri S, Wilding G, DeLeon O. Chest tube-delivered bupivacaine improves pain and decreases opioid use after thoracoscopy. Ann Thorac Surg. 2009;87(4):1040-1047. doi:10.1016/j.athoracsur.2008.12.099
17. Alex J, Ansari J, Bahalkar P, et al. Comparison of the immediate postoperative outcome of using the conventional two drains versus a single drain after lobectomy. Ann Thorac Surg. 2003;76(4):1046-1049. doi:10.1016/s0003-4975(03)00884-1
18. Gómez-Caro A, Roca MJ, Torres J, et al. Successful use of a single chest drain postlobectomy instead of two classical drains: a randomized study. Eur J Cardiothorac Surg. 2006;29(4):562-566. doi:10.1016/j.ejcts.2006.01.019
19. Okur E, Baysungur V, Tezel C, et al. Comparison of the single or double chest tube applications after pulmonary lobectomies. Eur J Cardiothorac Surg. 2009;35(1):32-36. doi:10.1016/j.ejcts.2008.09.009

**Figure 2. Flow chart of this parallel group randomised trial**


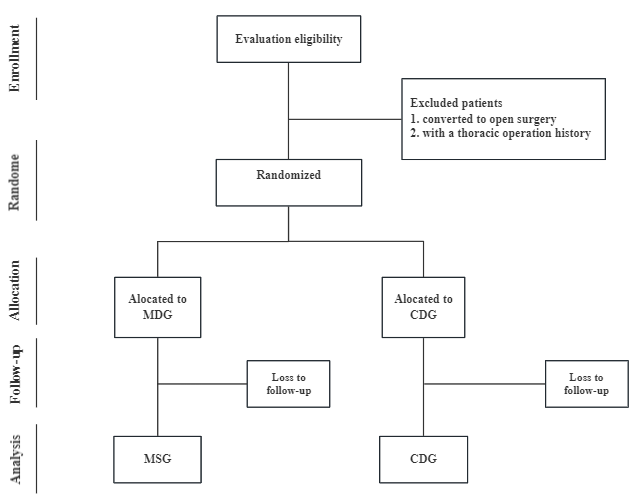


电视胸腔镜手术后多侧孔引流的安全性和可行性
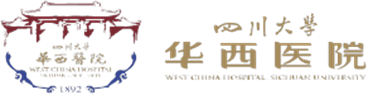
分析——一项随机对照研究

**Additional file 1**

Safety and feasibility of multi-lateral-pores drainage after video-assisted thoracoscopic surgery--A randomized controlled study

**填 表 人 Person filling in the form**

**开 始 日 期 Start date**

**截 至 日 期 Completed date**

**第一部分：患者基本信息**

**Part I: Basic information of patients**

| 住院号Hospital No. |  | 编号 No. | |  | 年龄 Age |  |
| --- | --- | --- | --- | --- | --- | --- |
| 姓名 Name |  | 性别 Gender | |  | 职业 Occupations |  |
| 居住地Current place of residence |  | | | | 身高 height | 体重 weight |
| 吸烟史 History of smoking |  | | | | | |
| 分组 Group |  | | | | | |
| 联系方式  Contact information | Tel：  Wechat： | | | | | |
| 相关疾病Comorbidity | 慢性疾病Chronic diseases：  胸部疾病手术史 Surgical history of chest disease： | | | | | |
| 入院日期  Date of admission |  | | 手术日期  Date of surgery |  | 出院日期  Date of discharge |  |
| 肺功能指标  Pulmonary function | FEV1/FVC: FEV1: FEV1%:  PEF（L/min）: DLco : DLco%: | | | | | |
| COPD | □ I级【FEV1≥80%】； □ II级【50%≤FEV1<80%】；  □ III级【30≤FEV1<50%】； □ IV级【FEV1<30%】 | | | | | |
| 出院诊断  Diagnosis at discharge | 主要诊断 Main diagnosis  次要诊断 Secondary Diagnoses | | | | | |
| 分期 Stage （9th） | □Ia □Ib □IIa □IIb □IIIa □IIIb □IIIc □IV | | | | | |
| 手 术 Surgical methods | □肺叶 Lobectomy； □肺段segmentectomy； □楔形Wedge resection | | | | | |
| ASA： | 手术时长Duration of surgery | | | |  | |
| 镇痛药物 Analgesics | 术中镇痛（药名/频次）Intraoperative analgesia was given (Drug name/frequency) | | | | | |
|  | 术后镇痛 （药名/频次） Postoperative analgesia (Drug name/frequency) | | | | | |
|  | 其他Others | | | | | |
| 病理Pathology | □腺癌AD □鳞癌SCC □其它other__ | | | | | |
| 病理亚型  Pathological Subtypes | □AAH □原位腺癌 □MIA  IA：□LPA □AC □PAP □MP □S □Mucinous □Other | | | | | |
| 备注 Note |  | | | | | |

**第二部分：研究部分**

**Part II: Research part**

| 引流表现 D***rainage performance*** | | | | |
| --- | --- | --- | --- | --- |
| 第一天引流量（ml） |  | 第二天引流量（ml） |  | |
| 第三天引流量（ml） |  | 第四天引流量（ml） | / | |
| 第五天引流量（ml） |  | 第六天引流量（ml） | |  |
| 平均每日引流量 Daily drainage volume（ml/d） | |  | | |
| 住院天数 Length of stay（d） | |  | | |
| 术后并发症 Postoperative complications | 1. □ 气胸 Pneumothorax 2. □ 胸腔积液 Pleural effusion 3. □ 皮下气肿 Subcutaneous emphysema 4. □ 声音嘶哑 Hoarseness 5. □ 肺部感染 Pulmonary infection 6. □ 持续性肺漏气 Prolonged air leak 7. □ 乳糜胸 Chylothorax 8. □ 声音嘶哑 Hemorrhage 9. □其他 Others | | | |
